# Microscopic thymoma with a thymic cyst that enlarged over a 2-year period

**DOI:** 10.1093/jscr/rjae038

**Published:** 2024-02-06

**Authors:** Hiroaki Komatsu, Nao Furukawa, Hirotaka Kinoshita, Kazunori Okabe

**Affiliations:** Department of Thoracic Surgery, Bell-Land General Hospital, Osaka 599-8247, Japan; Department of Thoracic Surgery, Bell-Land General Hospital, Osaka 599-8247, Japan; Department of Thoracic Surgery, Bell-Land General Hospital, Osaka 599-8247, Japan; Department of Thoracic Surgery, Bell-Land General Hospital, Osaka 599-8247, Japan

**Keywords:** microscopic thymoma, thymic cyst, robotic surgery

## Abstract

An 81-year-old woman was referred to our hospital because of right lung cancer. She underwent right upper lobectomy. Pathological examination revealed stage 1A adenocarcinoma. Four months postoperatively, chest computed tomography showed a small nodule with a diameter of 6 mm at the anterior mediastinum. After 2 years, the nodule had increased to 13 mm. To confirm the diagnosis and treat the mediastinal tumor, we resected the tumor and surrounding thymic tissue by a left robotic thoracic approach, considering the adhesion in the right thoracic cavity after right pulmonary resection. The operating time was 43 min. The patient had a favorable postoperative course and was discharged 3 days after surgery. Pathological examination revealed microscopic thymoma with a diameter of 400 μm very close to a thymic cyst. Microscopic thymoma can occur around a thymic cyst without myasthenia gravis, and the thymic tissue around the anterior mediastinal cyst should be resected.

## Introduction

Microscopic thymoma is defined as an epithelial proliferation smaller than 1 mm in diameter. It is usually multifocal and occurs in patients with myasthenia gravis (MG) [1].

We herein describe a patient who had microscopic thymoma with a thymic cyst that enlarged over a 2-year period.

## Case report

**Figure 1:**
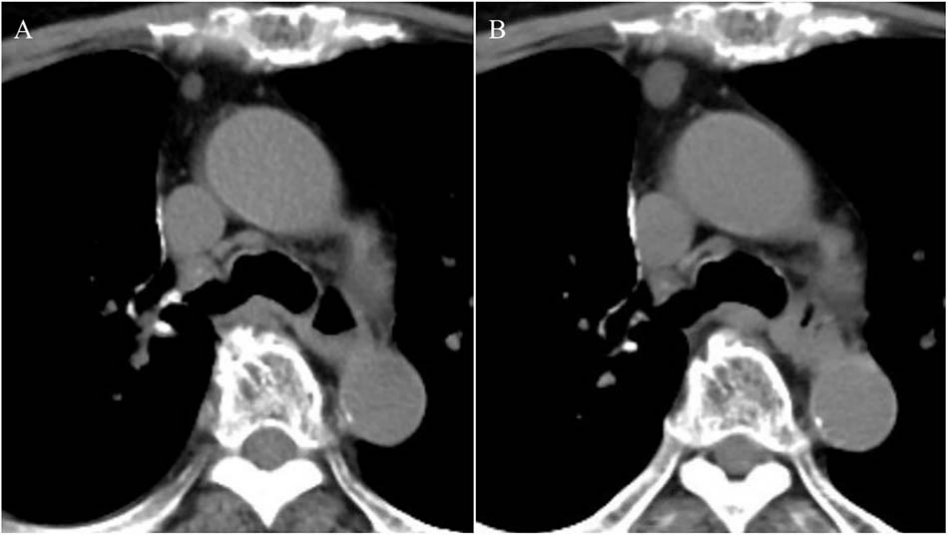
Chest computed tomography showing a small nodule with a diameter of 6 mm at the anterior mediastinum (A); after 2 years, the nodule had enlarged to 13 mm (B).

**Figure 2:**
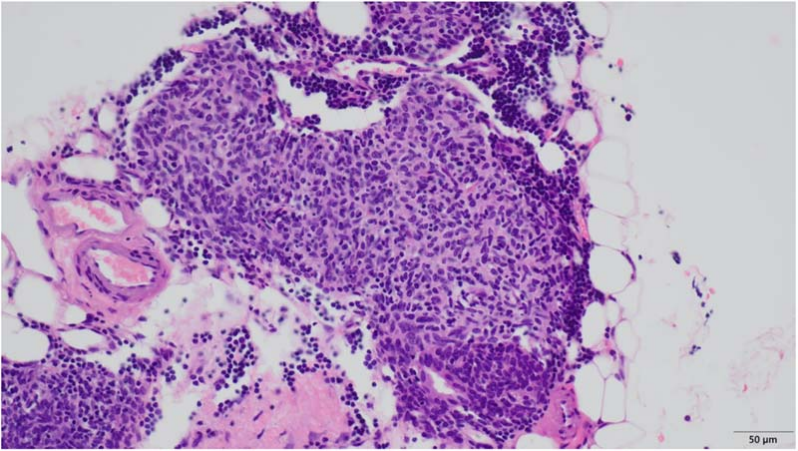
Microscopic findings revealed nodular hyperplasia of the thymic epithelium with a diameter of 400 μm.

An 81-year-old woman was referred to our hospital because of right lung cancer. She underwent right upper lobectomy by video-assisted thoracic surgery. Pathological examination revealed stage 1A adenocarcinoma. Four months postoperatively, chest computed tomography showed a small nodule with a diameter of 6 mm at the anterior mediastinum (Fig. 1A). After 2 years, the nodule had increased to 13 mm (Fig. 1B). Chest magnetic resonance imaging showed a mediastinal tumor with low-signal intensity on T1-weighted images and high-signal intensity on T2-weighted images, and it was diagnosed as a cystic tumor. For diagnosis and treatment, we resected the tumor and surrounding thymic tissue by a left robotic thoracic approach, considering the adhesion in the right thoracic cavity after right pulmonary resection. The operating time was 43 min, and the blood loss volume was 5 ml. The patient had a favorable postoperative course and was discharged 3 days after surgery. Pathological examination revealed a thymic cyst and microscopic thymoma with a diameter of 400 μm very close to the thymic cyst (Fig. 2).

## Discussion

Few reports have focused on microscopic thymoma without MG because microscopic thymoma has been incidentally found after thymectomy for MG [2]. Microscopic thymoma is considered a precursor of thymoma; however, some microscopic thymomas were reported as nodular hyperplasia of the thymic epithelium because the development of a thymoma from microscopic thymoma has not been proven [3].

Only one other report has described microscopic thymoma with a thymic cyst [2]. Although the patient initially underwent partial resection of the thymus with the thymic cyst, she additionally underwent completion thymectomy via median sternotomy. Additional resection was not performed in our patient because the surrounding thymic tissue was resected with the thymic cyst and there were no other microscopic thymoma lesions. Because other lesions may be present in the remaining thymus, careful follow-up is needed.

Our patient had some characteristics similar to the previous report. In both patients, the microscopic thymoma was very close to the thymic cyst, which enlarged over a 2-year period. These findings suggest an association between enlarging thymic cysts and microscopic thymomas. However, the microscopic thymoma did not arise in the wall of the thymic cyst, which differs from the thymomas in previous studies [4]. This does not suggest malignant potential of microscopic thymoma. Microscopic thymoma lacks morphological features of conventional thymomas, and ‘nodular hyperplasia of the thymic epithelium’ might be preferable to ‘microscopic thymoma’ [1].

We selected the left robotic thoracic approach, considering the adhesion in right thoracic cavity. Although the tumor was located near the right thoracic cavity, we safely and rapidly resected it from the narrow anterior mediastinum by taking advantage of robotic surgery, which provides free movement of robotic forceps under three-dimensional high-definition visualization [5]. Robotic surgery is useful for resection of an anterior mediastinal tumor located on the contralateral side of the thoracic approach.

Our study shows that microscopic thymoma can occur around a thymic cyst without MG. Although whether total thymectomy or additional resection is needed for microscopic thymoma remains controversial, thymic tissue around the anterior mediastinal cyst should be resected.
